# The Effects of Anti-Vaccine Conspiracy Theories on Vaccination Intentions

**DOI:** 10.1371/journal.pone.0089177

**Published:** 2014-02-20

**Authors:** Daniel Jolley, Karen M. Douglas

**Affiliations:** School of Psychology, University of Kent, Canterbury, United Kingdom; University of Georgia, United States of America

## Abstract

The current studies investigated the potential impact of anti-vaccine conspiracy beliefs, and exposure to anti-vaccine conspiracy theories, on vaccination intentions. In Study 1, British parents completed a questionnaire measuring beliefs in anti-vaccine conspiracy theories and the likelihood that they would have a fictitious child vaccinated. Results revealed a significant negative relationship between anti-vaccine conspiracy beliefs and vaccination intentions. This effect was mediated by the perceived dangers of vaccines, and feelings of powerlessness, disillusionment and mistrust in authorities. In Study 2, participants were exposed to information that either supported or refuted anti-vaccine conspiracy theories, or a control condition. Results revealed that participants who had been exposed to material supporting anti-vaccine conspiracy theories showed less intention to vaccinate than those in the anti-conspiracy condition or controls. This effect was mediated by the same variables as in Study 1. These findings point to the potentially detrimental consequences of anti-vaccine conspiracy theories, and highlight their potential role in shaping health-related behaviors.

## Introduction

The development of vaccines is one of the most important advances in the history of medicine, but in recent years, vaccination has declined in many regions of the world, especially in cases such as the combined Measles, Mumps and Rubella (MMR) vaccination [1]. One contributor to this particular decline appears to have been the publication of Andrew Wakefield’s article in *The Lancet* in 1998 concerning a possible link between the MMR vaccination and the appearance of autism [2], [3]. Although the article has since been retracted, the research discredited and the author is no longer permitted to practice medicine, lingering doubts persist and in many regions of the world, MMR vaccination rates lie well below the recommended 95% uptake [4]. In 2008, measles was declared to be endemic in the United Kingdom, 14 years after its spread was halted in the population [4]. Several methods have shown promising improvements in vaccination intentions generally, such as using expert sources to persuade people toward vaccination [5] and emphasizing that vaccination is normative [6]. However, one potential obstacle to such interventions may be the popularity of anti-vaccine conspiracy theories. The current research investigates the influence of such conspiracy allegations on vaccination intentions.

Conspiracy theories are attempts to explain events as the secret acts of powerful, malevolent forces [7], [8], [9], [10], [11]. For example, popular conspiracy theories allege that the 9/11 attacks were orchestrated by the US government, that Princess Diana was murdered by elements within the British establishment, and that the NASA moon landings were faked. Belief in conspiracy theories is widespread, with polls consistently indicating that more than 70% of Americans believe some form of conspiracy was responsible for President John F. Kennedy’s death [12]. Further, polls demonstrate that more than 20% of respondents endorse the idea that there is a link between childhood vaccines and autism [13]. Many other anti-vaccine conspiracy theories have emerged in recent years [14], [15]. At the heart of the anti-vaccine conspiracy movement lays the argument that large pharmaceutical companies and governments are covering up information about vaccines to meet their own sinister objectives. According to the most popular theories, pharmaceutical companies stand to make such healthy profits from vaccines that they bribe researchers to fake their data, cover up evidence of the harmful side effects of vaccines, and inflate statistics on vaccine efficacy [14], [15]. Anti-vaccine conspiracy theories therefore reflect suspicion and mistrust of scientific research examining vaccine efficacy and safety. Conspiracist ideation in general tends to be associated with a mistrust of science such as the rejection of climate science and other scientific propositions such as the link between smoking and lung cancer [16]. Along the same line, anti-vaccine conspiracy theories present an attempt to explain away overwhelming scientific evidence that vaccines are effective, safe, and necessary [17].

Although declining vaccination rates are undoubtedly a product of many contributing factors, it is important to consider the potential impact of conspiracy theories on vaccination intentions. In particular, parents who are faced with the decision to have their children vaccinated may be more likely to seek information about vaccines via the Internet than through their doctor [18]. Parents who go to the Internet will find that some of the top “hits” for vaccine-related search terms are websites that propagate anti-vaccine conspiracy theories [14], [15]. Although many people are skeptical of anti-vaccine conspiracy allegations, recent research suggests that such conspiracy theories tend to feature prominently in focus group discussions about vaccination [19].

Further, recent findings suggest that people tend to be persuaded by conspiracy theories they are exposed to without being aware of it [20]. Also, exposure to conspiracy theories has been found to have detrimental effects in other domains, such as reducing pro-environmental intentions and willingness to engage in politics [21], [22]. In the health domain, one prominent conspiracy theory proposes that birth control and HIV/AIDS are forms of genocide against the African American community. Endorsement of these conspiracy theories amongst African Americans has been found to be associated with negative attitudes towards contraceptive behaviors, which may potentially expose people to the risk of unwanted pregnancies and sexually transmitted illnesses [23], [24], [25]. Directly relevant to the current investigation, it has recently been shown that endorsement of a variety of unrelated conspiracy theories is associated with negative attitudes toward vaccination [26]. An emerging literature therefore points to the potential dangers of conspiracy theories. The current research explores the possibility that anti-vaccine conspiracy theories may present a significant obstacle to vaccine uptake.

In the current research, we also examine some of the potential factors that may mediate such effects. First, perceiving danger in vaccines tends to be associated with reluctance to vaccinate [27]. For example, many people believe that vaccines have dangerous side effects, and that exposure to the disease itself would often be preferable to the vaccination [4], [28]. Further, research suggests that perceived dangers play an important role in parental decisions to have their children vaccinated [29]. It is therefore possible that beliefs in conspiracy theories, or exposure to conspiracy theories, negatively influence people’s attitudes about the dangers of vaccines, and their subsequent decision to vaccinate. Feelings of powerlessness were measured as a second potential mediator, which refers to the perception of being incapable of influencing an outcome by taking action [30]. Research has demonstrated that powerlessness is associated with beliefs in conspiracy theories [22], [31] and also that feelings of political powerlessness mediate the relationship between exposure to conspiracy theories and voting intentions [22]. It is therefore possible that beliefs in anti-vaccine conspiracy theories, and exposure to such theories, increase feelings of powerlessness about the ability to change health outcomes, which subsequently reduce vaccination intentions.

Third, the current research examined the potential mediating role of disillusionment, or the feeling of disappointment that something is not what it was believed or hoped to be. Previous research has demonstrated that exposure to conspiracy theories increases political disillusionment [22], so it is reasonable to suppose that beliefs in anti-vaccine conspiracy theories or exposure to such theories may increase disillusionment with people responsible for the manufacture and administration of vaccines. This, in turn, may influence vaccination intentions. Finally, the current studies examined the potential mediating role of trust in authorities. Research has linked beliefs in conspiracy theories with low levels of trust [8], [33]. Further, distrust of medical information has been linked to reluctance to vaccinate [19]. Therefore, it is proposed here that beliefs in anti-vaccine conspiracy theories or exposure to such theories may decrease trust with medical officialdom and may, in turn, influence vaccination intentions.

In summary, the present research aims to explore the effect of anti-vaccine conspiracy beliefs on vaccination intentions. Two studies are presented, which test the predictions that belief in anti-vaccine conspiracy theories would be associated with decreased vaccination intentions (Study 1), and that exposure to anti-vaccine conspiracy theories would decrease vaccination intentions relative to an anti-conspiracy condition and control (Study 2). Both studies examined four potential mediators of the predicted effects.

## Study 1

The first study employed a correlational design where participants were asked to rate the extent to which they agreed or disagreed with statements related to a range of anti-vaccine conspiracy theories. Participants, who were all parents, were then presented with a scenario depicting a fictitious child. Here, they were asked to imagine that they were faced with the decision to have this child vaccinated against a specific (made up) disease. They were given some information about the disease and the vaccination and were asked to indicate their intention to have the child vaccinated.

### Methods

#### Ethics statement

The study was approved by the Psychology Ethics Committee at the University of Kent and all participants provided their written, informed consent.

#### Participants and design

Eighty-nine British parents (80 women and nine men, *M*_age_ = 38.06, *SD* = 9.25) participated in the study. The parents had an average of 1.35 (*SD* = .59) children, with the mean age of their youngest child being 3.38 (*SD* = 1.33). Participants were invited to take part in our study between September and December 2012 via poster advertisements, emails and via Facebook and Twitter where they were invited to complete an online questionnaire. They did so voluntarily and without incentive.

Anti-vaccine conspiracy beliefs were measured as the predictor variable and vaccination intentions as the criterion variable. Perceived dangers of vaccines, feelings of powerlessness, disillusionment, and trust in authorities were measured as potential mediators.

#### Materials and procedure

Participants indicated their informed consent before beginning the questionnaire. They were then asked to complete a scale measuring beliefs in anti-vaccine conspiracy theories. There were eight statements (e.g., “Vaccines are harmful, and this fact is covered up”; α = .85), where participants indicated their agreement on a seven-point scale in each case (1 = *strongly disagree, 7 = strongly agree*). The conspiracy belief scale from Study 1 is available in the Figure S2 in File S1.

Next, participants completed a scale measuring the perceived dangers of vaccines, adapted from existing materials [32]. There were eight statements (e.g., “Vaccines lead to allergies”, α = .86) where participants indicated their agreement on a seven-point scale (1 = *strongly disagree, 7 = strongly agree*). A three-item scale measuring a person’s feelings of powerlessness, specifically concerning vaccination was developed from previous research [22], [31]. Participants were asked to read the statements (e.g., “I feel that my actions will not stop the negative outcomes of immunizations”, α = .82) and rate their agreement on a six-point scale (*1 = strongly disagree, 6 = strongly agree*).

A scale was also included to measure participants’ feelings of disillusionment, specifically towards those involved in vaccinations (e.g., the government, pharmaceutical companies). This scale was adapted from existing materials [34], [22] and consisted of four statements (e.g., “I am very disappointed with those who are involved in immunizations (e.g., the government, pharmaceutical companies)”, α = .89) where participants indicated their agreement on a six-point scale (*1 = strongly disagree, 6 = strongly agree*). Further, trust towards authorities was measured by adapting items from existing scales (Leiserowitz (unpublished data) and [22]). There were two trust sources (corporations and government; Spearman-Brown Coefficient = .82), where participants indicated the extent to which they trusted the source to tell the truth about vaccination on a six-point scale (*1 = strongly distrust, 6 = strongly trust*). These four scales are available as Figure S1 in File S1. The order of measures was counterbalanced.

Finally, participants were asked to imagine a scenario in which they were the parent of an infant (Sophie, aged 8 months) [34], [35]. They were informed that their doctor had provided them with information regarding the (fictitious) disease *dysomeria*. Dysomeria was described as a DS-virus spread by droplet infection, which may lead to serious consequences with symptoms such as fever and vomiting. Participants were then informed about the vaccination against dysomeria, and that it is recommend by the U.S. Centers for Disease Control and Prevention (CDC) for people of all ages. After reading the scenario, participants were asked to indicate their intention to have the child vaccinated (“If you had the opportunity to vaccinate your child (Sophie, aged 8 months) against dysomeria next week, what would you decide?”). Participants indicated their intention on a seven-point scale (*1 = definitely not vaccinate, 7 = definitely vaccine*). At the conclusion of the study, participants were debriefed and were thanked for their participation.

### Results and Discussion

Raw data are available in File S2. For each variable, mean values were calculated by summing the individual scores and then dividing by the number of items. These mean scores were used in the statistical analyses. Descriptive statistics and correlations between variables are presented in Table 1. However, because the potential mediators were significantly correlated with each other, their factor structure was first examined via an exploratory factor analysis of the individual items using Varimax rotation. The same mediators were included in both Studies 1 and 2, so this analysis was conducted across data from both studies to increase power. The Kaiser-Meyer-Olkin (KMO) measure of sample adequacy was.93, exceeding the recommended value of.6 [36] and Bartlett’s Test of Sphericity [37] reached statistical significance, *X* ^2^ (136) = 4544.44, *p*<.001, indicating that the items had adequate common variance for factor analysis. Principal component analysis was then conducted, revealing four components with eigenvalues greater than 1 and extraction criterion of.30, explaining 52.5 per cent, 8.7 per cent, 8.7 per cent and 6.4 per cent of the variance respectively. The rotated solution revealed each component showing strong loadings, and all variables loading substantially on only one component. The results of this analysis therefore support the use of four separate mediators. The factor loadings for the mediators across both studies are available as Figure S1 in File S1.

**Table 1:** Intercorrelations and descriptive statistics between anti-vaccine conspiracy beliefs and vaccination intentions, and mediator variables.

	M (SD)	1	2	3	4	5	6
(1) Anti-vaccine conspiracy belief	2.00 (.89)	–	−.40***	.76***	.57***	.68***	−.46***
(2) Immunisation intention	5.63 (1.42)	−.40***	–	−.49***	.29**	−.36***	.20^¥^
(3) Dangers	2.97 (1.37)	.76***	−.48***	–	.58***	.60***	−.48***
(4) Powerlessness	3.16 (1.54)	.57***	.29**	.58***	–	.59***	−.31**
(5) Disillusionment	2.45 (1.40)	.68***	−.36***	.59***	.59***	–	−.41***
(6) Trust in authorities	3.09 (1.27)	−.46***	.20^¥^	−.48***	−.31**	−.41***	–

*Notes.* ^¥^ <.10. *p<.05. **p<. 01. ***p<. 001.

Participant age and gender were not associated with any of the potential mediators or the dependent measure and were therefore not analyzed further. As predicted, regression analyses revealed that anti-vaccine conspiracy beliefs were a significant negative predictor of vaccination intentions, *F*(1, 87) = 15.97, *p*<.001, *R^2^* = .16, *β* = −.63, *t* = − 3.10, *p*<. 001. Examining potential mediators of this effect, four separate regression analyses were conducted. As shown in Table 2, anti-vaccine conspiracy beliefs were a significant predictor of perceived dangers of vaccines, and feelings of powerlessness, disillusionment and trust in authorities, *F*(5, 83) = 120.37, *p*<.001, *R^2^* = .58; *F*(5, 83) = 41.70, *p*<.001, *R^2^* = .32; *F*(5, 83) = 74.43, *p*<.001, *R^2^* = .46; *F*(5, 83) = 23.00, *p*<.001, *R^2^* = .20, respectively.

**Table 2:** Four separate regressions examining anti-conspiracy belief as predictor, and four mediator variables as criterions.

	Criterion	*B*	*t*
1	Dangers	.76	10.98***
2	Powerlessness	.57	6.46***
3	Disillusionment	.68	8.63***
4	Trust in authorities	−.46	−4.80***

*Notes.* ***p<. 001.

To test the predicted pattern of mediation between anti-vaccine conspiracy beliefs and vaccination intentions, we used Hayes and Preacher’s [38] bootstrapping macro designed for SPSS to run a multiple mediation model. This method is a non-parametric test and therefore it does not violate assumptions of normality. The method is based on re-sampling a subset of the data many thousands of times, which subsequently creates a custom sampling distribution that is shaped like the data. This method encompasses two processes: first, the “direct effect” measures changes in the DV when the IV increases. In contrast, the “indirect effect” measures changes in the DV when the MV increases and the IV is fixed. The indirect effect is the test of mediation, and is our sole focus here. Bootstrapping therefore involves repeatedly estimating the indirect effect in each re-sampled data set. By repeating this process thousands of times, it builds an empirical approximation of the sampling distribution that constructs the confidence intervals [39]. In order to test the significance of the indirect effect, we used 5000 bootstrap re-samples to describe the confidence intervals of indirect effects in a manner that makes no assumptions about the distribution of the indirect effects.

**Figure 1:**
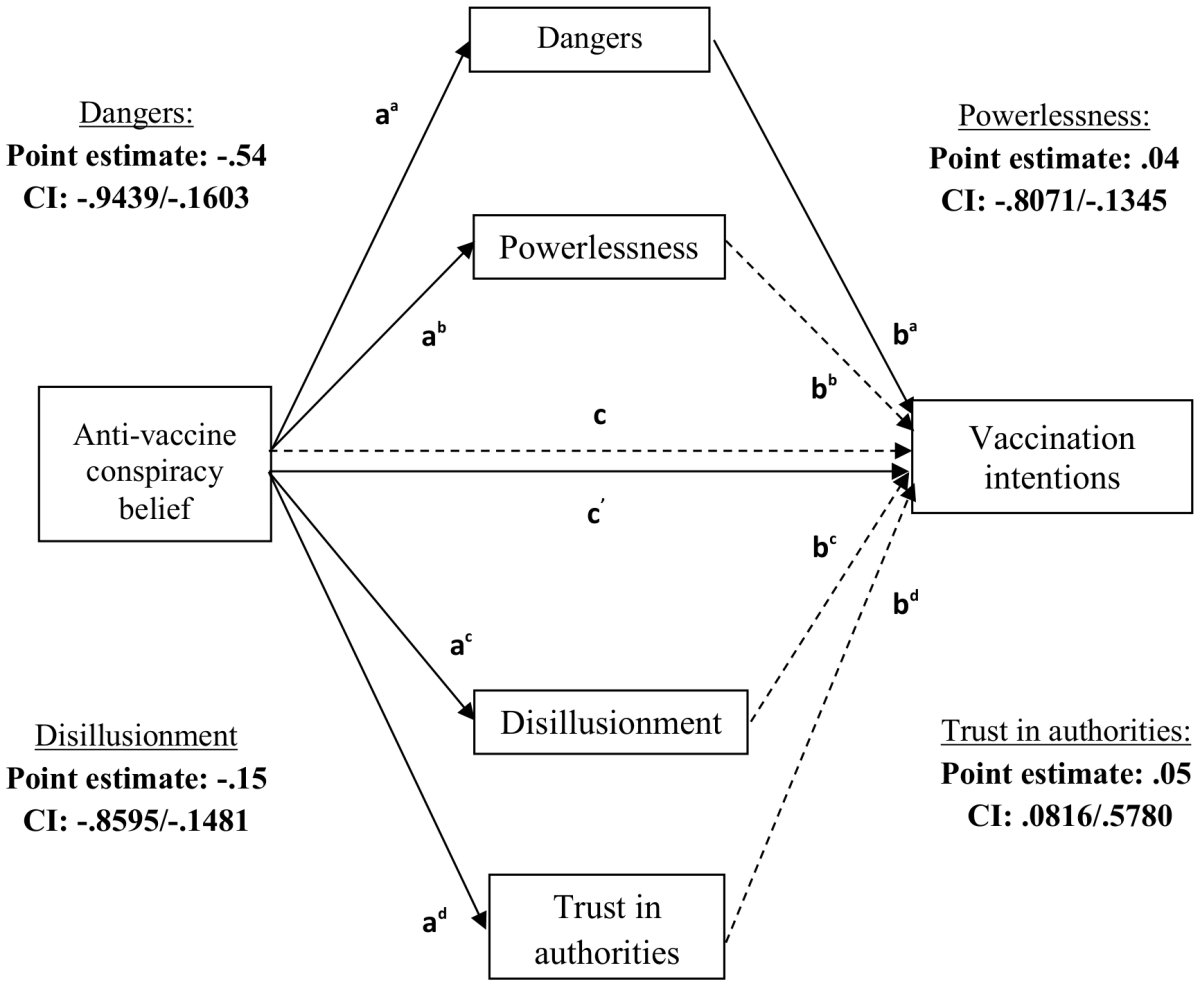
Multiple bootstrapping mediation test of the relationship between anti-vaccine conspiracy beliefs and vaccination intentions. Dashed lines highlight non-significant relationships and solid lines highlight significant relationships. Boldface type highlights a significant effect as determined by the Monte Carlo 90% confidence interval (CI) which does not contain a zero.

As argued by Hayes [40], an indirect effect is estimated as being significant if the confidence intervals do not contain a zero, as opposed to significance in the individual paths. This is because the mediation model is not pertinent to whether the individual paths (“a” path (IV to mediator), “b” path (mediator to DV, controlling for the IV), “c” path (IV to DV) or “c′” path (IV to DV, controlling for the mediators)) are either significant or non-significant. Results from the current study are presented in Table 3 and Figure 1. The multiple mediation analysis of the effect of anti-vaccine conspiracy beliefs on vaccination intentions indicated that perceived dangers of vaccines and feelings of powerlessness, disillusionment and trust in authorities (controlling for each other) were each significant mediators of this effect.

**Table 3:** Multiple bootstrapping mediation test of the relationship between anti-vaccine conspiracy beliefs and vaccination intentions.

	Normal test theory				
Mediator (*MV*)		Dependent (*DV*)			
Path	Coeff. (s.e.)	Path	Coeff. (s.e.)	Path	Coeff. (s.e.)
a^a^	1.17 (.11)***	c	−.63 (.16)***	c′	−.02 (.26)
a^b^	.97 (.15)***				
a^c^	1.06 (.12)***				
a^d^	−.65 (.14)***				
‘M*V*’				b^a^	−.46 (.16)***
				b^b^	.04 (.12)
				b^c^	−.14 (.14)
				b^d^	.08 (.13)

*Note*. ***p<.01.

A mediation test of the relationship between anti-vaccine conspiracy beliefs (IV; a) and vaccination intentions (DV; c) through perceived dangers of vaccines ^(a)^, and feelings of powerlessness ^(b)^, disillusionment ^(c)^ and trust in authorities ^(d)^ (MVs; b) (N = 89; 5000 bootstrap samples).

Therefore, as hypothesized, anti-vaccine conspiracy beliefs predicted vaccination intentions. Participants who endorsed anti-vaccine conspiracy theories to a greater extent indicated less intention to vaccinate. Further, anti-vaccine conspiracy beliefs were associated with three potential mediator variables that had been examined in previous research [22] and also the perceived dangers of vaccines. When all factors were taken into account, each was a significant mediator of the relationship between anti-vaccine conspiracy beliefs and vaccination intentions. Using an experimental design, Study 2 was designed to replicate and extend these findings by investigating the casual relationship between anti-vaccine conspiracy theories and vaccination intentions, via perceived dangers of vaccines, and feelings of powerlessness, disillusionment and mistrust in authorities.

## Study 2

In Study 2, participants were exposed to material supporting anti-vaccine conspiracy theories (versus anti-conspiracy material, or a control condition). Participants were then asked to indicate their intention to have a fictitious child vaccinated as in Study 1. It was predicted that exposure to material supporting anti-vaccine conspiracy theories would negatively influence vaccination intentions, compared to the other conditions. The potential mediators examined in Study 1 were also measured. It was predicted that all variables would be associated with vaccination intentions, and that each would mediate the effect of exposure to conspiracy theories on vaccination intentions.

### Methods

#### Ethics statement

The study was approved by the Psychology Ethics Committee at the University of Kent and all participants provided their written, informed consent.

#### Participants and design

Two hundred forty six participants (146 women and 100 men, *M*_age_ = 34.76, *SD* = 12.90) were recruited in April 2013 via Amazon’s Mechanical Turk (MTurk). Participants were residents of the U.S.A. and received 70 cents in exchange for their participation. MTurk is an online crowdsourcing tool for collecting high-quality, inexpensive experimental data and it is widely used in psychological research [41], [42]. Researchers have found MTurk workers to be at least as representative of the U.S. population as traditional internet subject pools, with gender, race, age, and education matching the population more closely than internet samples in general [42].

Two questions randomly placed within the questionnaire (e.g., “So we can be sure that you are reading the questions carefully, please answer “Strongly disagree” to this question”) were included to identify participants who had rushed the questionnaire. Further, a timer was used to identify participants who had spent less than 30 seconds reading the vaccine-related material and who had thus exceeded reading speed capabilities for upper college students [43]. Participants who failed the screening were removed from analyses (26 participants from the pro-conspiracy condition, 19 from the anti-conspiracy condition and 13 from the control condition). The final sample size used for data analysis was therefore 188 (112 women and 76 men, *M*_age_ = 36.33, *SD* = 13.40). There were 60 participants in the pro-conspiracy condition, 62 in the anti-conspiracy condition, and 66 in the control condition. Within the final sample, 83 (44.15%) were parents, who had an average of 1.30 (*SD*:535) children, with the youngest being 4.37 (*SD* = 1.10) years old.

A single-factor independent variable (pro-conspiracy vs. anti-conspiracy vs. control) between-subject design was employed. A manipulation check measured participants’ judgements that a series of anti-vaccine conspiracy theories are true. As in Study 1, participants reported the perceived dangers of vaccines, and feelings of powerlessness, disillusionment, and trust in authorities. Finally, participants were again asked to indicate their intention to have a fictional child vaccinated.

#### Materials and procedure

As in Study 1, this was an online questionnaire in which participants were first asked to give their informed consent. Next, participants were either exposed to information that supported anti-vaccine conspiracy theories (pro-conspiracy condition) or information that refuted conspiracy theories (anti-conspiracy condition). A control condition was also included, where no further information was given. Participants were randomly assigned to one of the three conditions. The pro-conspiracy article began by arguing that people within the vaccine industry are guilty of misrepresenting data. It then continued to provide specific examples such as the idea that hiding information about vaccines is purely motivated by profit and there is significant evidence that vaccines hurt more than they help. An extract from the pro-conspiracy article was as follows:

*“…further, there is a significant amount of evidence that vaccines can hurt more than they help. For example, by the year 2002, tens of thousands of reactions to vaccines, including deaths, were reported. One must magnify these figures tenfold, because it is estimated that 90% of doctors do not report incidents…”.*

The anti-conspiracy article differed by arguing that there are no reasons to doubt the efficacy and safety of vaccines. It then continued to provide specific examples such as the idea that the financial benefits of preventing illnesses far outweigh the profits made from vaccines and that there is little evidence to suggest that vaccines are harmful. An extract was as follows:

*“…further, there is little evidence to suggest that vaccines are harmful. The side effects are minimal and whilst millions of people have been immunised over the years, less than.005% have ever had an adverse reaction to a vaccine…”.*

The manipulation is available as Figure S3 and S4 in File S1. The term ‘conspiracy theory’ was not mentioned in either of the articles. To check that the manipulation was successful, participants rated the likelihood that a series of anti-vaccine conspiracy theories are true. Those in the control condition also completed this manipulation check. There were eleven statements in total (e.g., “*Misrepresentation of the efficacy of vaccines is motivated by profit*”, α = .88), where participants indicated their agreement on a seven-point scale (*1 = strongly disagree, 7 = strongly agree*). The manipulation check is available as Figure S5 in File S1. Participants then indicated their perceived dangers of vaccines (α = .90), and feelings of powerlessness (α = .88), disillusionment (α = .93) and trust in authorities (Spearman-Brown Coefficient = .73). The order of measures was counterbalanced. Participants next read the scenario as in Study 1 and indicated their intention to have a fictional child vaccinated against a made up disease. At the end of the study, participants were told that the information presented in the article was fictional, and was written for the purposes of the study. Participants were also pointed towards websites containing factual information about vaccines, vaccine efficacy and vaccine safety before being thanked and paid for their participation.

### Results and Discussion

Raw data are available in File S2. For each variable, mean values were calculated by summing the individual scores and then dividing by the number of items. These mean scores were used in the statistical analyses. None of the analyses were affected by the participants’ status as parents or non-parents, nor their age or gender. These variables were therefore not analyzed further.

#### Manipulation check

There was a significant difference in endorsement of anti-vaccine conspiracy theories between conditions, *F*(2, 185) = 13.79, *p<*. 001, *η^2^* = .15. Endorsement of anti-vaccine conspiracy theories was significantly higher in the pro-conspiracy condition (*M = *4.11, *SD* = 1.41) than the anti-conspiracy condition (*M = *2.93, *SD* = 1.14, *p*<.001) and the control condition (*M = *3.56, *SD* = 1.21, *p = *.014). The manipulation was therefore successful. Endorsement of anti-vaccine conspiracy theories was significantly lower in the anti-conspiracy condition than the control condition (*p* = .005). Because the anti-conspiracy condition reduced conspiracy beliefs below baseline, we report analyses comparing the pro-conspiracy condition to both the anti-conspiracy and control conditions.

#### Anti-vaccine conspiracy theories and vaccination intentions

As hypothesized, results revealed a significant difference in vaccination intentions across conditions, *F*(2, 185) = 4.81, *p* = .009, *η^2^* = .05. Vaccination intentions were significantly lower in the pro-conspiracy condition (*M = *4.87, *SD* = 1.74) than the anti-conspiracy condition (*M = *5.69, *SD* = 1.31, *p* = .003) and the control condition (*M = *5.47, *SD* = 1.50, *p = *.028). Intentions were not significantly different between the anti-conspiracy condition and control (*p* = .407).

#### Testing mediation

To test potential mediators of this effect, separate ANOVAs were firstly conducted with conspiracy condition (pro-conspiracy versus anti-conspiracy versus control) as the independent variable, and summed scores on all potential mediators (perceived vaccine dangers, powerlessness, disillusionment and trust in authorities) as dependent variables. Results revealed a significant difference in perceived dangers of vaccines between conditions, *F*(2, 185) = 7.61, *p* = .001, *η^2^* = .08. Perceived dangers were higher in the pro-conspiracy condition (*M = *4.00, *SD* = 1.46) than the anti-conspiracy condition (*M = *2.97, *SD* = 1.42, *p*<.001) and the control condition (*M = *2.39, *SD* = 1.71, *p = *.021). Perceived dangers were not significantly different between the anti-conspiracy and control conditions (*p* = .11).

Results also revealed a significant difference in powerlessness between conditions, *F*(2, 185) = 3.44, *p* = .034, *η^2^* = .04. Powerlessness was significantly higher in the pro-conspiracy condition (*M = *4.25, *SD* = 1.43) than the anti-conspiracy condition (*M = *3.46, *SD* = 1.78, *p* = .008). Powerlessness was not significantly different between the pro-conspiracy and control conditions (*p* = .097), and the anti-conspiracy and control conditions (*p* = .327). There was a significant difference in disillusionment between conditions, *F*(2, 185) = 7.46, *p* = .001, *η^2^* = .08. Disillusionment was significantly higher in the pro-conspiracy condition (*M = *3.65, *SD* = 1.71) than the anti-conspiracy condition (*M = *2.52, *SD* = 1.78, *p*<.001). However, disillusionment was not significantly higher than the control (*M = *3.11, *SD* = 1.55, *p = *.062). Disillusionment was significantly lower in the anti-conspiracy condition relative to the control condition (*p* = .041).

Finally, results revealed no significant difference in trust in authorities between conditions, *F*(2, 185) = 2.32, *p* = .10, *η^2^* = .03. However, trust was significantly lower in the pro-conspiracy condition (*M = *2.60, *SD* = 1.01) than the control condition (*M = *2.97, *SD* = 1.04, *p = *.048). Trust was not significantly lower in the pro-conspiracy condition relative to the anti-conspiracy condition (*M = *2.66, *SD* = .1.07, *p* = .745), or anti-conspiracy and control (*p* = .095).

**Figure 2:**
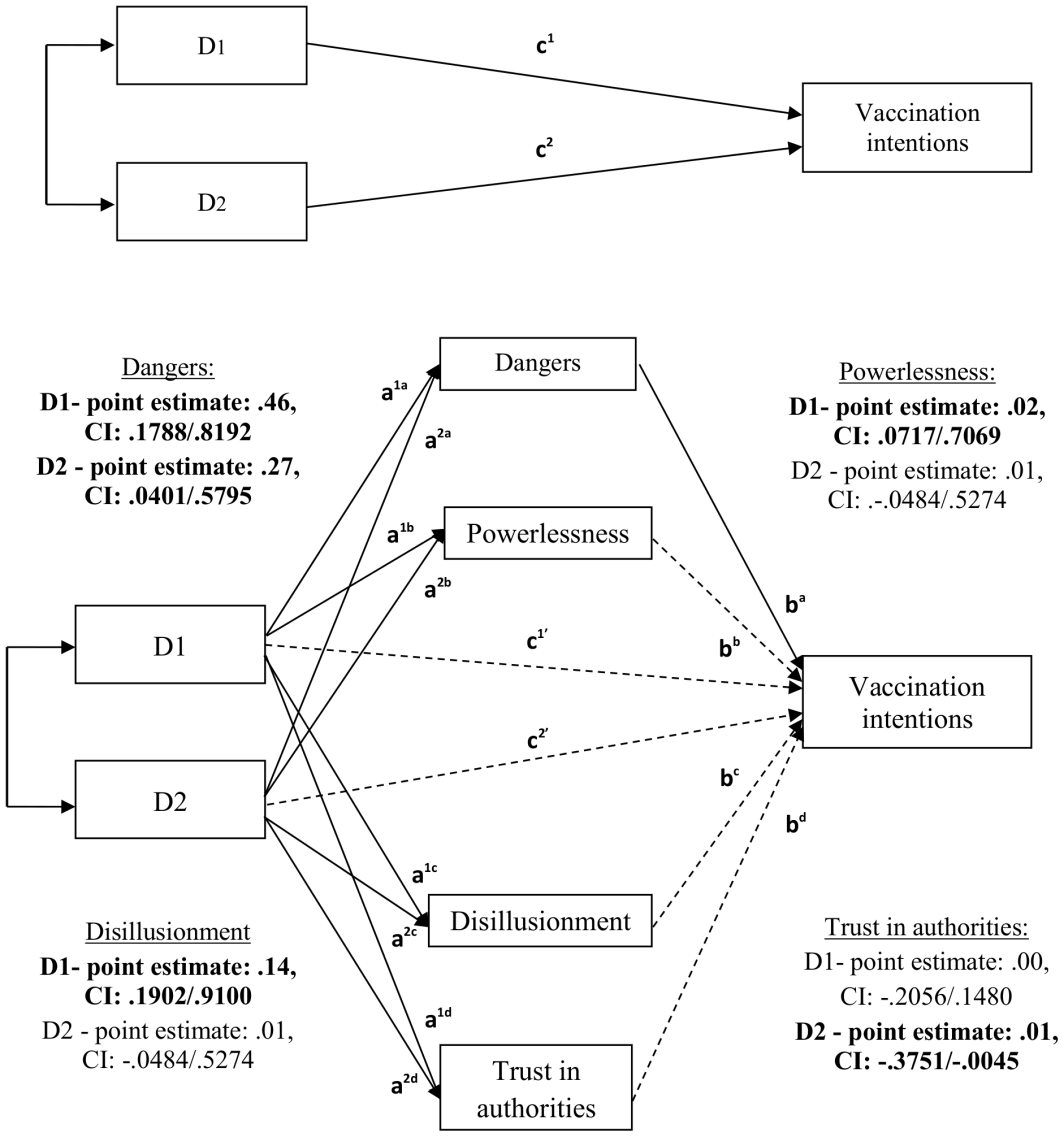
Multiple mediation test between conspiracy condition (using indicate coding) and vaccination intentions. Dashed straight lines highlight non-significant path relationships and solid straight lines highlight significant path relationships. Boldface type highlights a significant effect as determined by the Monte Carlo 95% confidence interval (CI) which does not contain a zero.

Each of the candidate mediators was then examined in a test of mediation in order to explain the effect of the conspiracy conditions (pro-conspiracy versus anti-conspiracy, versus control) on vaccination intentions. This was carried out using Hayes and Preacher’s [38] bootstrapping method for indirect effects, as in Study 1. However, the method differed slightly, allowing mediations between the three conspiracy conditions to be tested by the use of indicator coding. This was done using Hayes and Preacher’s [38] SPSS mediate macro. The pro-conspiracy condition was coded as the representative condition and was compared to the anti-conspiracy condition (D1) and control (D2) separately. The SPSS macro had one indicator variable (D1, pro-conspiracy versus anti-conspiracy) as the IV, and the other as a covariate (D2, pro-conspiracy versus control), before simultaneously swapping the variables around to complete the second meditational analysis (D2, pro-conspiracy versus control as the IV and D1, pro-conspiracy versus anti-conspiracy as the covariate). This allows the mediational models to be tested whilst controlling for the effect of the parallel analysis, which is completed automatically by the SPSS macro. As in Study 1, an indirect effect is then estimated as being significant from the confidence intervals not containing a zero, as opposed to significance in the individual paths [40]. Results are presented in Table 4 and Figure 2.

**Table 4:** Multiple mediation test of conspiracy condition (using indicate coding) on vaccination intentions.

	Normal test theory					
	Mediator (*MV*)		Dependant (*DV*)			
IndictorCoding	Path	Coeff. (s.e.)	Path	Coeff. (s.e.)	Path	Coeff. (s.e.)
D1	a^1a^	−1.13 (.29)***	c^1^	.83 (.28)***	c^1^′	.24 (.25)
	a^1b^	−.79 (.30)**				
	a^1c^	−1.13 (.30)***				
	a^1d^	.06 (.19)				
D2	a^2a^	−.61 (.26)**	c^2^	.60 (.27)**	c^2^′	.27 (.24)
	a^2b^	.37 (.19)**				
	a^2c^	−.48 (.30)				
	a^2d^	.37 (.19)*^¥^*				
	‘M*V*’				b^a^	−.45 (.11)***
					b^b^	.02 (.07)
					b^c^	−.13 (.10)
					b^d^	.01 (.10)
						

*Note*. *^¥^p<.*10. **p<.05. ***p<.01.

A mediation test of conspiracy condition (D1, pro-conspiracy versus anti-conspiracy, versus D2, pro-conspiracy versus control) on vaccination intentions (DV) through perceived dangers of vaccines ^(a)^, and feelings of powerlessness ^(b)^, disillusionment ^(c)^ and trust in authorities ^(d)^ (MVs) (N = 188; 5000 bootstrap samples).

The multiple mediation analysis of the effect of pro-conspiracy versus anti-conspiracy condition on vaccination intentions (D1) (when controlling for pro-conspiracy versus control, D2) indicated that perceived vaccine dangers, and feelings of powerlessness and disillusionment (controlling for all mediators) were mediators of this effect. Second, the effect for D2 (controlling for D1) indicated that perceived vaccine dangers and trust in authorities (controlling for all mediators) significantly mediated this effect.

Therefore, as expected, participants who were exposed to material supporting anti-vaccine conspiracy theories showed reluctance to have a child vaccinated compared to the other two conditions. The perceived dangers of vaccines were a consistent mediator across conditions. Further, feelings of powerlessness and disillusionment mediated the difference between the pro- and anti-conspiracy conditions, and mistrust in authorities mediated the difference between the pro-conspiracy and control conditions.

## General Discussion

The current research suggests that anti-vaccine conspiracy theories may have more than a trivial effect on vaccination intentions. In two studies, it has been demonstrated that beliefs in anti-vaccine conspiracy theories – such as the belief that research on vaccine efficacy is manipulated to make profits for pharmaceutical companies – are associated with reduced vaccination intentions. Further, the current research has demonstrated that exposure to anti-vaccine conspiracy theories directly affects vaccination intentions. Both effects were significantly mediated by the perceived dangers of vaccines. In Study 1, the effect was further mediated by feelings of powerlessness, disillusionment and mistrust in authorities. In Study 2, feelings of powerlessness and disillusionment mediated the difference between the pro- and anti-conspiracy conditions, and mistrust in authorities mediated the difference between the pro-conspiracy and control conditions. Therefore, overall, anti-vaccine conspiracy theories appear to introduce undue suspicion about vaccine safety, and increase feelings of powerlessness and disillusionment, whilst decreasing trust in authorities, which in turn introduce reluctance to vaccinate. This work demonstrates empirically, and to our knowledge for the first time, that anti-vaccine conspiracy theories may therefore present an obstacle to vaccine uptake.

Although a variety of attempts to increase vaccination intentions have shown promising success in recent years [5], [6], the current research suggests that future attempts to intervene on vaccine reluctance should also consider the role of conspiracy theorizing. Specifically, because beliefs in conspiracy theories in general are associated with a mistrust of scientific claims [16], interventions that cite claims by scientists and medical professionals may also meet with suspicion. Such attempts at intervention may therefore fail on people who are sympathetic to a variety of conspiracy claims [14], [15].

Instead, successful interventions may focus on direct counter-arguments against the conspiracy allegations themselves [44]. Indeed, the finding here that the anti-conspiracy condition – which directly refuted conspiracy allegations – reduced conspiracy beliefs below baseline, suggests that this may be a promising avenue for intervention. This could be further investigated by manipulating the source of the information presenting the counter-arguments against conspiracy allegations (e.g., governmental bodies, independent vaccine agencies, academic researchers). However, it is important to note that whilst the anti-conspiracy condition reduced conspiracy beliefs below baseline, this was not associated with increased intentions to vaccinate. This may be consistent with the argument that misinformation tends to be resistant to correction [45]. That is, once the very idea of a conspiracy has been mentioned and has taken root, even strong counter-arguments may be unable to lead to behavioral action. Future research may therefore also consider the impact of the order in which misinformation and counter-arguments are presented. Further, future research may investigate the role of prior warnings and the continued influence of misinformation on behavioural intentions [46]. Nevertheless, it is argued here that future interventions to increase vaccine uptake should address the impact of anti-vaccine conspiracy theories.

The current research had some important limitations that should also be addressed in future research. First, it is important to note that although the effects observed throughout this research were statistically robust, the effects sizes were small (e.g., *η^2^* = .05 for the effect of vaccine information on vaccination intentions in Study 2). This means that the proportion of variance in vaccine intentions explained by exposure to conspiracy theories was quite modest and there are potentially many other factors that contribute to vaccine intentions. Nonetheless, small reductions in uptake, especially in cases such as the MMR vaccine, can have large effects since the recommended uptake to ensure herd immunity is 95% [1].

It should also be noted that endorsement of anti-vaccine conspiracy theories tended to be around or below the midpoint, except in the condition where participants were exposed to anti-vaccine conspiracy information (*M* = 4.11 on a 7-point scale in Study 2). Therefore, the participants were not strong endorsers of anti-vaccine conspiracy theories, meaning that different patterns of findings may emerge for those who do strongly endorse conspiracy theories. Similarly, different strategies for successful intervention may apply for people who hold strong anti-vaccine conspiracy beliefs than those who do not hold strong beliefs [44]. Future research could consider these possibilities.

Further, the pattern of mediation is less clear in Study 2 than in Study 1 and future research may endeavour to uncover additional mediators or isolate one key mediator of the conspiracy-vaccination intention link. However, the current research has identified a number of factors that are influenced by exposure to conspiracy theories, which, in turn, influence vaccination intentions. Finally, the findings were based on self-report intentions to have a fictional child vaccinated against a made up disease. As is well known, intentions do not always translate into behaviors [47], [48], [49]. Future research may therefore examine associations between anti-vaccine conspiracy beliefs and actual vaccination behavior. Future research could also examine larger samples and potentially identify the impact of conspiracy theories in geographical areas that have dangerously low vaccination uptake.

Future research may also focus on the individual difference characteristics that pre-dispose individuals to anti-vaccine conspiracy beliefs. Psychologists are learning more about the traits and characteristics associated with beliefs in conspiracy theories more generally, such as mistrust, anomie, political cynicism and Machiavellianism [8], [20], [31], [50], and it will be useful to know if the same, or different factors predict anti-vaccine conspiracy beliefs. Further, another avenue for intervening on vaccination reluctance may be to focus on individuals who possess the personal characteristics that make them receptive to conspiracy claims. Theorists [44] note the possibility of directing anti-conspiracy information at potential consumers of conspiracy theories, in order to “inoculate” them against accepting such theories, and a method like this may also be effective in encouraging people to reject anti-vaccine conspiracy claims and promoting vaccine uptake.

In conclusion, the current research suggests that anti-vaccine conspiracy theories may have significant and detrimental consequences. Specifically, they appear to reduce vaccination intentions by inducing undue concern about the dangers of vaccines, and increasing powerlessness, disillusionment, and mistrust. This research is timely in the face of declining vaccination rates, and recent outbreaks of vaccinated-against diseases such as measles. Indeed, at the time of writing this article, 1,325 people in Wales had contracted measles, and medical officials were becoming increasingly concerned about vaccination uptake in general across the United Kingdom [51]. The current research also speaks to a broader concern about conspiracy theorizing and science denial [8], [16], [44]. Ongoing investigations are needed to further identify the social consequences of conspiracism, and to identify potential ways to combat the effects of an ever-growing culture of conspiracism.

## Supporting Information

File S1 Contains Figure S1 (Items and factor loadings of the four mediator variables), Figure S2 (Anti-vaccine conspiracy belief scale used in Study 1), Figure S3 (Pro-conspiracy manipulation excerpt used in Study 2), Figure S4 (Anti-conspiracy manipulation excerpt used in Study 2), Figure S5 (Anti-vaccine conspiracy theory manipulation check used in Study 2).
(DOCX)

File S2 Raw data from Study 1 and Study 2.
(XLS)
