# Supplementary material for: The Effects of Anti-Vaccine Conspiracy Theories on Vaccination Intentions
Source: PLoS One. 2014 Feb 20;9(2):e89177. doi: 10.1371/journal.pone.0089177 (PMC3930676; doi:10.1371/journal.pone.0089177)
Supplement: File S1 — Contains Figure S1 (Items and factor loadings of the four mediator variables), Figure S2 (Anti-vaccine conspiracy belief scale used in Study 1), Figure S3 (Pro-conspiracy manipulation excerpt used in Study 2), Figure S4 (Anti-conspiracy manipulation excerpt used in Study 2), Figure S5 (Anti-vaccine conspiracy theory manipulation check used in Study 2). (DOCX) [file pone.0089177.s001.docx]

**Supplementary materials**

**The effects of anti-vaccine conspiracy theories on vaccination intentions**

**Figure S1**

| **Items** | **1**  **Perceived dangers** | **2**  **Powerlessness** | **3 Disillusionment** | **4**  **Trust in authorities** |
| --- | --- | --- | --- | --- |
| I feel uncertain about the potential side-effects of immunizations. | .84 |  |  |  |
| I feel uncertain about the safety of immunizations. | .83 |  |  |  |
| A large number of early vaccinations expose an infant s immune system to avoidable risks. | .74 |  |  |  |
| Multiple vaccines overwhelm the infant’s immune system. | .74 |  |  |  |
| The side-effects of vaccinations are unforeseeable. | .71 |  |  |  |
| Vaccines lead to allergies. | .67 |  |  |  |
| I feel uncertain about the motives of those involved in immunizations (governments, pharmaceutical companies etc.). | .61 |  |  |  |
| Vaccinations cause the illnesses they are intended to protect against. | .61 |  |  |  |
| I feel that immunization concerns are too big for my actions to have an impact. |  | .84 |  |  |
| I feel that my actions will not stop the negative outcomes of immunizations. |  | .84 |  |  |
| When it comes to immunizations, I feel powerless. |  | .83 |  |  |
| I have given up on those who are involved in immunizations (e.g., the government, pharmaceutical companies, etc.). |  |  | .85 |  |
| Those who are involved in immunizations (e.g., the government, pharmaceutical companies, etc.) are no longer important to me as they used to be. |  |  | .83 |  |
| I feel tricked, cheated or deceived by those who are involved in immunizations (e.g., the government, pharmaceutical companies, etc.) |  |  | .77 |  |
| I am very disappointed with those who are involved in immunizations (e.g., the government, pharmaceutical companies, etc.) |  |  | .71 |  |
| Corporations |  |  |  | .91 |
| National government |  |  |  | .88 |

*Items and factor loadings of the four mediator variables*

**Figure S2**

*Anti-vaccine conspiracy belief scale used in Study 1*

| **Question number** | **Item** |
| --- | --- |
| 1 | Immunizations allow governments to track and control people. |
| 2 | Vaccines are harmful, and this fact is covered up. |
| 3 | Tiny devices are placed in vaccines to track people. |
| 4 | Pharmaceutical companies, scientists and academics work together to cover up the dangers of vaccines. |
| 5 | Vaccines are not tampered with. * |
| 6 | The government is trying to cover up the link between vaccines and autism. |
| 7 | Tiny devices are implanted in vaccines for use in mind control experiments. |
| 8 | The flu vaccine allows the government to monitor the elderly through the implantation of tiny tracking devices. |

** Item reverse-scored.*

**Figure S3**

*Pro-conspiracy manipulation excerpt used in Study 2*

**Please read this short excerpt from a recent Internet article about vaccines. We will ask you some questions about the excerpt later in the study, so please read it carefully.**

Should we be suspicious of vaccines? Should we consider the proposal that those in power, whether governments or pharmaceutical companies, hide crucial information about vaccines from the public?

Several specific questions have been raised about vaccines. For example, are people within the industry faking data on vaccine efficacy? Do vaccines hurt more than they help? Is the industry deceiving people purely to make a profit?

Questions such as these are widespread in the media and on the Internet, but should we pay any attention to them?

The answer is YES. There are many reasons to think twice about vaccines.

For example, people within the vaccine industry are guilty of misrepresenting data on the efficacy of vaccines. Evidence suggests that diseases such as smallpox and paralytic polio have not been eradicated by vaccines. They have simple been renamed and these diseases still exist among the population.

Further, there is a significant amount of evidence that vaccines can hurt more than they help. For example, by the year 2002, tens of thousands of reactions to vaccines, including deaths, were reported. One must magnify these figures tenfold, because it is estimated that 90% of doctors do not report incidents.

Perhaps unsurprisingly, therefore, a recent news poll of 1,024 adults showed that an overwhelming majority of 61% believed that vaccines were harmful and a further 17% were unsure. Only 22% of respondents believed the official account that vaccines are safe.

Hiding information about vaccines is purely motivated by profit. The increase in government recommended vaccines for children has more than doubled since 1985, making pharmaceutical companies very wealthy. The profit margins made by pharmaceutical companies are extremely high. According to market research, vaccine sales will more than double this year, from $19 billion in 2012 to $39 billion in 2013. This is nearly five times the $8 billion in vaccine sales in 2004.

There are other reasons to doubt the efficacy and safety of vaccines… [*article continues]*

**Figure S4**

*Anti –conspiracy manipulation excerpt used in Study 2*

**Please read this short excerpt from a recent Internet article about vaccines. We will ask you some questions about the excerpt later in the study, so please read it carefully.**

Should we be suspicious of vaccines? Should we consider the proposal that those in power, whether governments or pharmaceutical companies, hide crucial information about vaccines from the public?

Several specific questions have been raised about vaccines. For example, are people within the industry faking data on vaccine efficacy? Do vaccines hurt more than they help? Is the industry deceiving people purely to make a profit?

Questions such as these are widespread in the media and on the Internet, but should we pay any attention to them?

The answer is NO. There is no reason to think twice about vaccines.

For example, there is convincing and accurate evidence for the success of vaccines. Diseases such as smallpox and paralytic polio have been completely eradicated by vaccines. These once fatal diseases no longer exist among the population.

Further, there is little evidence to suggest that vaccines are harmful. The side effects are minimal and whilst millions of people have been immunized over the years, less than .005% have ever had an adverse reaction to a vaccine.

Perhaps unsurprisingly, therefore, a recent news poll of 1,024 adults showed that an overwhelming majority of 61% believed that vaccines are safe and only a further 17% were unsure. Only 22% of respondents believed that vaccines were harmful and unsafe.

The financial benefits of preventing illnesses far outweigh the profits made from vaccines by pharmaceutical companies. For example, in 2001, routine childhood immunization in the USA was estimated to save over $40 billion per birth-year cohort in overall social costs including $10 billion in direct health costs. The government recommends vaccines for children to improve public health and save money, not to make a profit.

There are other reasons to doubt the efficacy and safety of vaccines… [*article continues]*

**Figure S5**

*Anti-vaccine conspiracy theory manipulation check used in Study 2*

| **Question number** | **Item** |
| --- | --- |
| 1 | Many diseases, said to have been eradicated by vaccines, are still around today. |
| 2 | Misrepresentation of the efficacy of vaccines is motivated by profit. |
| 3 | Vaccines are harmful, and this fact is covered up. |
| 4 | Vaccine safety data is often fabricated. |
| 5 | Immunizing children is harmful and this fact is covered up. |
| 6 | People are deceived about vaccine safety. |
| 7 | Pharmaceutical companies cover up the dangers of vaccines. |
| 8 | People are deceived about vaccine efficacy. |
| 9 | Vaccines are not harmful. * |
| 10 | Vaccine efficacy data is often fabricated. |
| 11 | People are deceived about vaccine safety. |

** Item reverse-scored.*
